# Clinical and Gait Parameters Related to Pelvic Retraction in Patients with Spastic Hemiplegia

**DOI:** 10.3390/jcm8050679

**Published:** 2019-05-14

**Authors:** Kun-Bo Park, Hoon Park, Byoung Kyu Park, Sharkawy Wagih Abdel-Baki, Hyun Woo Kim

**Affiliations:** 1 Division of Pediatric Orthopaedic Surgery, Severance Children’s Hospital, Yonsei University College of Medicine, 50-1 Yonsei-ro, Seodaemun-gu, Seoul 03722, Korea; pedoskbp@yuhs.ac (K.-B.P.); Sharkawy_ortho@hotmail.com (S.W.A.-B.); 2 Department of Orthopaedic Surgery, Gangnam Severance Hospital, Seoul 06273, Korea; hoondeng@yuhs.ac; 3 Department of Orthopaedic Surgery, Inje University Haeundae Paik Hospital, Busan 48108, Korea; yspbk@naver.com

**Keywords:** pelvic retraction, hemiplegia, cerebral palsy

## Abstract

Pelvic retraction during walking is a common finding seen in patients with spastic hemiplegia. However, potential factors related to this condition have not been comprehensively examined in a systemic manner in previous studies. The purpose of this study was to elucidate any clinical and gait parameters related to pelvic retraction in patients with hemiplegic cerebral palsy. A total of 212 independent ambulatory patients were enrolled in the study. Group I consisted of 113 patients who had persistent pelvic retraction, and Group II of 99 with a normal range of pelvic rotation throughout the gait cycle as evidenced by kinematic analysis. A multivariate logistic regression analysis using a clustering technique was performed, with use of eight gait factors and five clinical factors. Decreased ankle dorsiflexion, increased hip internal rotation, increased anterior pelvic tilt, the Winters classification type II, and *asymmetrical posturing of the upper extremity during gait* were found to be related to pelvic retraction. This is the only study including a broader array of assessment domains of both clinical and gait parameters with a considerably large and homogenous population with hemiplegia. Further studies will be needed to see whether the rectification of those parameters may improve abnormal gait and pelvic retraction in hemiplegia.

## 1. Introduction

During normal gait, the ipsilateral pelvis is internally rotated at the initial foot contact owing to forward positioning of the foot, and then there is a continuous external rotation of the pelvis to an extent of less than 5 degrees by the time of contralateral foot contact [1]. This normal pelvic movement can be compromised in patients with underlying pathologic conditions. Pelvic retraction, excessive external rotation of the pelvis during gait, is a common finding seen in patients with cerebral palsy [2]. It produces functional problems and cosmetic concerns due to an asymmetric gait.

Pelvic retraction has been considered a consequence of primary neurological deficit *per se*, resulting from a lesion of the central nervous system [3,4]. However, it may be secondary to muscle spasticity or may be a coping response to the torsional deformity of the lower extremity. O’Sullivan et al. examined average and maximum values of gait parameters and found that ankle plantarflexor tightness, increased hip flexion and hip internal rotation were the significant features seen in patients with cerebral palsy and pelvic retraction [2]. On the contrary, contractures of hip and knee flexors were reported to be factors associated with pelvic retraction in another study [5]. Several surgical procedures have also been introduced to reduce pelvic retraction [6,7]; some improvements of pelvic retraction after derotational osteotomy of the femur were observed, and an increased hip internal rotation associated with an increased femoral anteversion was suggested to be the contributing factor for pelvic retraction in hemiplegia [8,9].

However, a review of the literature is difficult because heterogeneous groups of patients with hemiplegia and diplegia were included in most studies. To identify any factors related to pelvic retraction in cerebral palsy, the inclusion of a large patient cohort with homogeneous disease entity is the first requirement as patients with spastic hemiplegia and diplegia show different gait patterns. Furthermore, the definition of pelvic retraction was inconstant among studies and many authors simply used the average or maximum value of pelvic rotation in order to examine the degrees of external rotation of the pelvis and to determine pelvic retraction; however, those values cannot represent real persistent pelvic retraction during the entire phases of gait and patients with exaggerated pelvic motion may be misinterpreted as having pelvic retraction [10,11]. 

For better prediction of treatment outcomes, not only the three-dimensional gait parameters but also the clinical features related to abnormal gait pattern should be considered [8,12,13,14,15]. Nonetheless, there have been more endeavours at the interpretation and biomechanical analysis of abnormal pelvic motions with small subgroups of patients enrolled in each study [2,5,7,16]. Also, it is necessary to investigate the effects of unilateral involvement of the entire limbs and the severity of clinical deficits of the ipsilateral limbs on the development of pelvic retraction. The purpose of this present study is to identify any clinical features and gait parameters that may be related to pelvic retraction in patients with spastic hemiplegic cerebral palsy (SHCP). To the best of our knowledge, this is the only study including a broader array of assessment domains of both clinical and gait parameters with a considerably large and homogenous population with hemiplegia.

## 2. Experimental Section

This was a retrospective study and was approved by our hospital’s institutional review board (IRB No. 4-2009-0035). Written informed consent was obtained from all participants’ parents. The research methods were carried out in accordance with the relevant institutional guidelines and regulations.

### 2.1. Subjects

**Figure 1:**
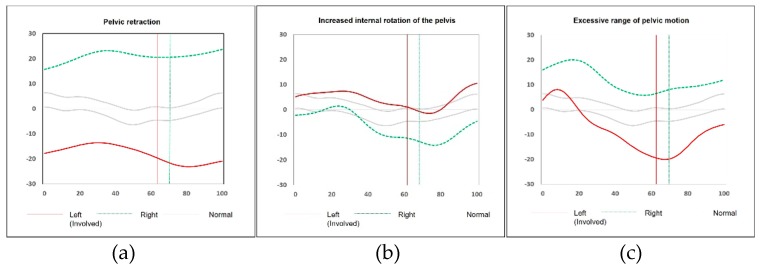
Demonstration of three types of pelvic rotation which may be seen in a patient with left hemiplegia. (**a**) Pelvic retraction: the average degree of external pelvic rotation on the left side (red line) is 15.64°, and the consistently increased external rotation of the pelvis throughout the gait cycle is more than two standard deviations from the norm. (**b**) Increased internal rotation of the pelvis compared to the contralateral right side (green line): the average degree of internal pelvic rotation on the left side is 4.91° which is within normal range. (**c**) Excessive range of pelvic motion: the average degree of external pelvic rotation on the left side is 6.05°; however, increased internal rotation at early stance phase followed by a sinusoidal type of pelvic rotation are noted. Patients with an increased internal rotation of the pelvis or an excessive range of pelvic motion were excluded in this study.

Three hundred and twelve independent ambulatory patients with SHCP (Gross Motor Function Classification System, GMFCS I or II) who underwent three-dimensional instrumented gait analysis (VICON 370 Motion Analysis System, Oxford Metrics, Oxford, England) were recruited. First, we observed the pattern of pelvic rotation using each patient’s kinematic graphs, and the presence of a pelvic retraction was defined as when the consistently increased external rotation of the pelvis throughout the gait cycle (Figure 1a) was more than two standard deviations (SDs) from our normative database (<−4.75°) [2,5]. Second, patients with increased internal rotation of the involved side compared to the normal contralateral side (Figure 1b) were excluded as this represents primary external pelvic rotation on the contralateral side [1,11]. Also, patients with an excessive or exaggerated range of pelvic motion characterized by an increased internal rotation of the pelvis at early stance phase and then followed by a sinusoidal type of pelvic rotation (Figure 1c) were excluded; this represents compensation for a reduced sagittal plane excursion of motion [11]. The degree of pelvic rotation was calculated by averaging the maximum and the minimum values of pelvic rotation in all patients [2]. 

**Figure 2:**
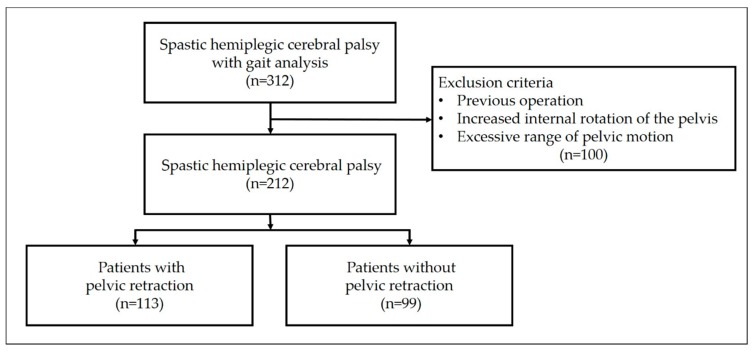
Flow diagram of inclusion and exclusion criteria in the study.

In total, 212 patients were enrolled in the study (Figure 2). There were 84 (39.6%) females and 128 (60.4%) males. The average age at the time of gait analysis was eight years and eight months (range, 38 months to 29 years). One hundred and twenty-seven patients (59.9%) had right hemiplegia, and 85 (40.1%) had left hemiplegia. The subjects were divided into two groups; Group I consisted of 113 patients (53.3%) who had pelvic retraction, and Group II comprised 99 patients (46.7%) who did not have pelvic retraction.

### 2.2. Clinical and Gait Parameters 

A thorough physical examination was performed in all patients: modified Ashworth scale [17], passive range of motion in the hip, knee, and ankle joints; popliteal angle for hamstring tightness, Duncan-Ely test for rectus femoris spasticity or tightness, and ankle dorsiflexion angle during the Silfverskiöld test for the detection of gastrocnemius/Achilles tendon tightness. The degree of ankle dorsiflexion was measured with the knee flexed and extended. Limb length discrepancy was assessed by measuring the distance from the anterior superior iliac spine to the medial malleolus in both sides in all patients. A scanogram was also checked in 175 (82.5%) patients (93 patients in Group I and 82 in Group II). Femoral anteversion was measured by the trochanteric palpation method [18] and tibial torsion was measured using the bimalleolar axis in all patients [19]. One hundred and seventy-two patients (81.1%) also underwent a computed tomographic (CT) scan to measure both femoral anteversion and tibial torsion.

Gait analysis was performed using a VICON 370 Motion Analysis System (Oxford Metrics, Oxford, England) with six infrared cameras and a Helen Hayes marker set. Data on ground–reaction forces were gathered from multiple force platforms (Advanced Mechanical Technology, Watertown, MA, USA). All subjects were asked to walk barefoot at a self-selected speed along a 15-m walkway with markers in place. We selected values at the initial contact, maximum, minimum, and average values for each kinematic parameter at each phase of gait. Several variables that have been regarded as clinically unimportant were excluded; furthermore, those variables have been known to be interrelated with each other in a complex way, and may act as confounding factors in the interpretation of gait data and in understanding abnormal movement [2,5,9,14,20,21,22].

**Figure 3:**
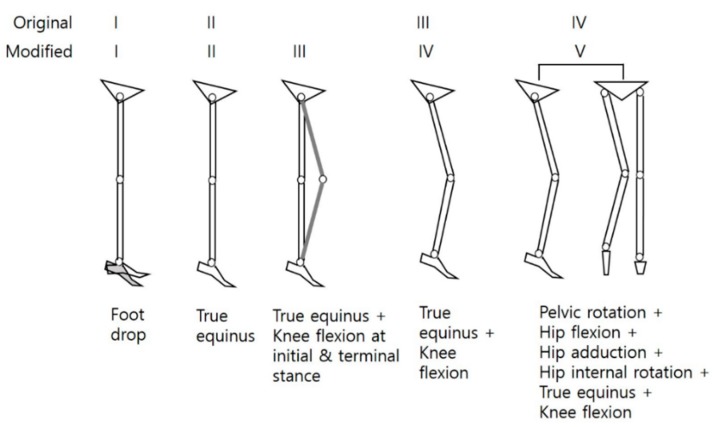
The Winters classification (modified and reproduced) [23,26].

**Figure 4:**
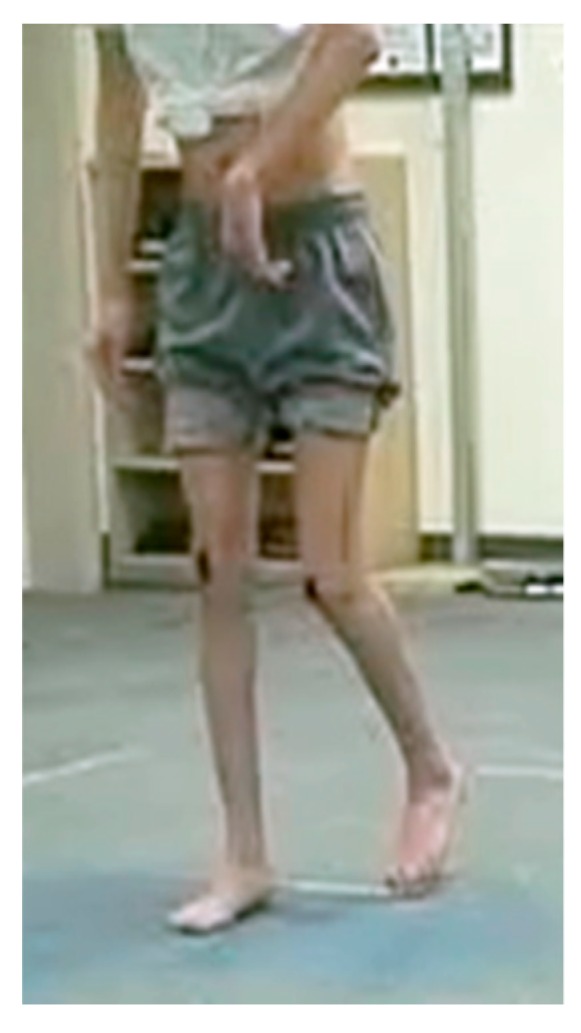
A hemiplegic patient with asymmetrical posturing of the upper extremity during walking.

Two pediatric orthopaedic surgeons reviewed all the videotaping for visual gait analysis, three-dimensional gait analysis data, dynamic foot-pressure measurements (pedobarographs), gross clinical photos, and standing plain radiographs of the lower extremity in all patients. The presence of an equinovarus foot deformity was defined as when inversion and plantar flexion of the hindfoot were present in standing position and a significant pressure exerted on the lateral forefoot and midfoot compared to the non-involved were confirmed on the dynamic pedobarographs (Tekscan, South Boston, MA, USA). The determination of knee recurvatum gait and the type according to the classification by Winters et al. [23] were done using gait data and visual analysis. We referred recurvatum gait as a clinical static variable as it is a descriptive type of qualitative classification. We modified the original Winters classification as follows; a patient with a normal range of knee motion but having increased knee flexion at initial contact and terminal stance phase of gait was categorized into type III (Figure 3). Consequently, patients with the original type III with increased knee flexion and type IV with increased hip flexion, hip internal rotation, and pelvic retraction were then re-classified as type IV and type V, respectively. The presence of an asymmetrical posturing of the upper extremity was defined as when the patient has more than 30 degrees of elbow flexion contractures and typical posturing becomes apparent when the patient walks (Figure 4) [24,25]. 

### 2.3. Statistical Analyses

Statistical analyses were performed using IBM^®^SPSS^®^ software version 23 (IBM Corporation, Armonk, NY, USA). The level of significance was set at *p* < 0.05. An independent t-test was used for initial comparison between the groups. Twenty-one gait parameters were selected for the clustering technique analysis: range of pelvic motion in the sagittal plane, average anterior pelvic tilt, range of pelvic motion in the coronal plane, average pelvic obliquity, minimum hip sagittal angle, average hip sagittal angle, maximum hip coronal angle, average hip coronal angle, maximum hip transverse angle, average hip transverse angle, minimum knee sagittal angle, range of knee motion in sagittal plane, knee sagittal angle at initial contact, average knee transverse angle, maximum ankle sagittal angle, minimum ankle sagittal angle, average ankle sagittal angle, ankle sagittal angle at initial contact, average ankle transverse angle, maximum foot progression angle, and average foot progression angle. With six temporospatial parameters, a total of 27 gait parameters were clustered as gait factors, with the consideration of the differences in patients’ ages and the correlation between the gait parameters [2,13,14,22,27]. 

Finally, eight gait factors (ankle dorsiflexion, temporospatial parameter (walking speed, stride & step length), temporospatial parameter (cadence, stride & step time), internal rotation of foot and ankle, knee extension, pelvic obliquity and hip abduction, anterior pelvic tilt, hip internal rotation) and five clinical factors (modified Winters classification, Achilles tendon tightness, gastrocnemius tightness, pes equinovarus, asymmetrical posturing of the upper extremity) were included for the multivariate logistic regression analysis.

## 3. Results

### 3.1. Comparisons between the Groups (Group I vs Group II) 

Patients (7.94 ± 4.49 years) in the group of pelvic retraction (Group I) were younger than those (9.49 ± 5.06 years) in the group of normal range of pelvic rotation (Group II) (*p* = 0.0189). There were no statistical differences between the groups in terms of limb length discrepancy, femoral anteversion, and tibial torsion. Gastrocnemius and Achilles tendon tightness were more frequent in Group II (Table 1). Pes equinovarus, asymmetrical posturing of the upper extremity, and severe types of modified Winters classification were encountered more frequently in Group I. However, there was no difference in the existence of recurvatum gait between the groups. Modified Winters types IV and V were seen in small numbers of the total study population, affecting 36 (17.0%) and 6 (2.8%) patients, respectively; and types II, IV, and V were more common in Group I, and type I was more common in Group II (Table 2).

The stride length and step length were found to be shortened in Group I, and the walking speed was reduced in Group I (Table 3). In the sagittal plane, the average anterior pelvic tilt was increased in Group I compared to Group II. Maximum hip extension, maximum knee flexion, and ankle dorsiflexion were found to be reduced in Group I. At the initial foot contact, ankle dorsiflexion was decreased more in Group I. In the coronal plane, patients in Group I had more downward pelvic obliquity during the stance phase. Average hip rotation showed a greater internal rotation in Group I, and average ankle rotation showed a greater internal rotation in Group I (Table 4).

### 3.2. Gait Factors Classified by Clustering Technique

The first eight factors accounted for 79.96% of the total variability between the groups. The first factor mainly indicated ankle dorsiflexion. The second was a function of step/stride length and walking speed, while the third consisted of cadence, step, and stride time. The fourth corresponded to the internal rotation of the foot and ankle, and the fifth was the knee extension. The sixth was the pelvic obliquity and hip abduction, the seventh was anterior pelvic tilt, and the eighth was the hip internal rotation (Table 5). 

### 3.3. Multivariate Logistic Regression Analysis

The multivariate logistic regression analysis with eight gait factors and five clinical factors revealed that factor 1 (ankle dorsiflexion) and factors 7 and 8 (anterior pelvic tilt and hip internal rotation) have a relationship with pelvic retraction. In the modified Winters classification, only types II and V showed a relation with pelvic retraction. Asymmetrical posturing of the upper extremity was related to pelvic retraction. Gastrocnemius and Achilles tendon tightness were not significant variables, despite factor 1 (ankle dorsiflexion) having a relationship with pelvic retraction (Table 6).

## 4. Discussion 

Previous studies suggested several associated factors related to pelvic retraction during gait in cerebral palsy. However, the results of single event multi-level surgery performed in patients with cerebral palsy were not consistent in terms of the improvement of pelvic retraction [6,7,20,21,30]. Furthermore, pelvic retraction observed to be improved at the short-term follow-up was not noted to be maintained at the long-term follow-up [2,9,16,20]. These findings suggest that other factors rather than those addressed in previous studies must have existed. In the present study, we have found that pelvic retraction in spastic hemiplegia is related to the following parameters: increased anterior pelvic tilt and hip internal rotation; decreased ankle dorsiflexion; type II according to the classification by Winters et al.; and asymmetrical posturing of the affected upper extremity during gait. If any factors related to pelvic retraction could be clarified before the operation, the surgical results may be predicted more reliably.

The patients with a “relative” internal rotation of the pelvis on the affected side caused by primary external pelvic rotation on the contralateral side, and the patients with an excessive range of pelvic motion were not included in the present study. Only the patients with an excessive external rotation of the pelvis outside two standard deviations throughout the gait cycle were defined as having pelvic retraction. The determination of pelvic retraction should not be made by a simple measurement of the average values of the external rotation of the pelvis [1,10]. Not only the average values of the parameters but also the “patterns” of pelvic rotation throughout the gait cycle should be considered, as described earlier in Section 2.1. 

The transverse motion of the hip joint during gait is affected by the torsional deformity of the femur and/or tibia as well as by the spastic muscles. Another concern is whether the correction of an increased internal rotation of the hip may also treat pelvic retraction. Improvements in pelvic rotation after femoral derotational osteotomy have been reported, however there was no differentiation between the hemiplegic and diplegic patients in their studies [7,20]. Furthermore, the number of each subgroup of the patients enrolled was too small to draw a definitive conclusion [8,27]. Rutz et al. observed an improvement in pelvic rotation and a correction of hip internal rotation only in patients with Winters classification type IV; they had had soft tissue releases of the hip flexors and adductors in addition to femoral derotational osteotomy [31]. It is our opinion that to determine the effects of derotation osteotomy on the improvement of pelvic retraction, those factors found in our study as well as any abnormal movement occurring in the non-involved side of limb should be considered as well.

Decreased ankle dorsiflexion found in patients with Group I was due to tightness of the gastrocnemius and Achilles tendon, and this was confirmed by comparing the physical examination findings between the groups. Normal forward progression of the tibia over the supporting foot during the stance phase of gait is prevented by tight calf muscles [2,5,7,30]. Pelvic retraction might occur as a consequence of insufficient forward progression of the body during the stance phase, and the lengthening of shortened ankle plantarflexors would improve pelvic retraction [6]. However, we could not appreciate any other effects of tight gastrocnemius and Achilles tendon, as these clinical features were not statistically significant in the multivariate logistic regression analysis.

On the contrary to the previous observations [2], our results showed that there is no relationship between pelvic retraction and tightness/spasticity of the rectus femoris. Despite no difference in the rectus tightness, patients in Group I had lower degrees of hip extension and had decreased maximum knee flexion during gait compared to Group II. The rectus femoris tightness has been thought to be secondary to longstanding inappropriate hip extension, and this diminished range of hip extension is related to ankle equinus [2]. Decreased ankle dorsiflexion may cause reduced hip extension and may also elicit decreased knee flexion. On the other hand, reduced hip and knee motions in the sagittal plane have been known to be associated with an excessive range of pelvic motion [1]. It is our opinion that there may be differences in terms of the degree of spasticity of the rectus femoris or the amount of reduced range of motion, compared to the patients with excessive pelvic motions.

Some previous studies introduced modified Winters classifications [26,32] in order to allocate subsets of patients that cannot be classified with the original system, and to make up the limitations in classifying types I, II, and III which were determined only by abnormalities in the sagittal plane. We also adapted a modified Winters classification in the study because 20% of our patients had increased knee flexion at initial and terminal stance phasse of gait, as well as having a normal range of knee motion in the sagittal plane; they were categorized into modified type III, and patients with original type III with increased knee flexion were classified as modified type IV. The characteristic finding of modified types III and IV was knee flexion compared to type II which has only ankle equinus. In our logistic regression analysis, allocation into type II was found to be related to pelvic retraction. Increased knee flexion is the characteristic finding in modified types III and IV; this increased knee flexion may act as a compensation for forward progression of the tibia. We think that pelvic retraction may decrease with forward progression of the knee. Patients with modified type V in our series showed less pelvic retraction than patients with type I. However, as only six patients (2.8%) were modified type V compared to 47 patients with type I (22.2%), it may be difficult to conclude that patients with modified type V have less risk of pelvic retraction. 

No previous studies mentioned an association between asymmetrical posturing of the upper extremity during walking and pelvic retraction. Asymmetrical posturing was more frequent in the group with pelvic retraction, and was also found to be a related factor to pelvic retraction in the logistic regression analysis. Nevertheless, it is not certain that any surgeries on the upper extremity may improve pelvic retraction as the asymmetrical posturing itself is related not only to the trunk balance during gait but also to the severity of a primary neurologic impairment per se [33]. 

Our study has several limitations: we selected only five clinical factors, however those clinical factors have traditionally been frequent in patients with hemiplegia and have also been considered as significant factors related to the abnormal gait; scanogram and CT scan were not performed in about 20% of our series as they refused radiological exposure. Nonetheless, the trochanteric palpation method is a useful and reliable method to measure femoral anteversion in patients with cerebral palsy [18], and the use of the bimalleolar axis for tibial torsion shows a high correlation with CT measurement [19]. Furthermore, there were no significant differences in both physical examination and radiographic evaluation between the groups; and as the clinically perceived pelvic retraction may be a movement that may result from complex movements occurring in the sagittal, coronal, or transverse plane, the clinician should be aware that there may be differences between the real gait and three-dimensional gait analysis. 

## 5. Conclusions

To the best of our knowledge, this is the only study including a broader array of assessment domains of both clinical and gait parameters with a considerably large and homogenous population with hemiplegia. We conclude that pelvic retraction is more likely in patients with decreased ankle dorsiflexion, increased hip internal rotation, and increased anterior pelvic tilt. However, clinical features such as Winters classification type II and asymmetrical posturing are also frequently seen in patients with pelvic retraction. Further studies will be needed to see if the rectification of those parameters may improve abnormal gait and pelvic retraction in spastic hemiplegia.

## Figures and Tables

**Table 1:** Comparisons of clinical parameters between the groups.

	Group I	Group II	*p
Limb length discrepancy, mm	6.8 ± 7.0	6.7 ± 7.2	0.881
Limb length discrepancy (by scanogram), mm	6.61 ± 4.67	7.16 ± 6.30	0.521
Femoral anteversion, degrees	28.8 ± 11.7	26.6 ± 13.0	0.182
Femoral anteversion (by CT) ^(1)^, degrees	29.25 ± 11.88	26.22 ± 12.43	0.105
Tibial torsion, degrees	29.4 ± 12.1	26.5 ± 9.4	0.061
Tibial torsion (by CT) ^(2)^, degrees	29.09 ± 10.54	27.51 ± 10.00	0.371
Hip flexion, degrees	120.78 ± 5.55	120.43 ± 2.41	0.565
Hip external rotation, degrees	45.37 ± 9.96	48.12 ± 13.32	0.159
Hip internal rotation, degrees	42.26 ± 9.79	42.39 ± 12.88	0.943
Popliteal angle, degrees	21.49 ± 13.85	21.55 ± 12.33	0.977
Rectus femoris tightness, grade	0(32), 1(24), 2(3), 3(0)	0(43), 1(16), 2(2), 3(1)	0.153
Gastrocnemius tightness, degrees	83.87 ± 17.15	90.64 ± 8.34	<0.001 *
Achilles tendon tightness, degrees	91.03 ± 17.30	98.35 ± 9.29	<0.001 *

Values are given as mean ± standard deviation. *p < 0.05. ^(1)^ Measured by CT scan. The normal value is 9.3 ± 8.6 degrees for male and 14.8 ± 9.1 for female [28]. ^(2)^ Measured by CT scan. The normal value is 37.8 ± 7.3 degrees [29].

**Table 2:** Comparisons of observational gait parameters between the groups.

	Group I	Group II	*p
Recurvatum gait	Yes (50) *vs.* No (63)	Yes (35) *vs.* No (64)	0.187
Pes equinovarus	Yes (44) *vs.* No (68)	Yes (23) *vs.* No (76)	0.028 *
Asymmetrical posturing of the upper extremity	Yes (50) *vs.* No (63)	Yes (50) *vs.* No (63)	<0.001 *
Modified Winters classification	I(17) II(45) III(23) IV(24) V(4)	I(34) II(30) III(21) IV(12) V(2)	0.011 *

*p < 0.05.

**Table 3:** Comparisons of temporospatial parameters between the groups.

	Group I	Group II	*p
Cadence, steps/min	123.81 ± 26.33	123.86 ± 19.64	0.987
Step time, s	0.54 ± 0.17	0.51 ± 0.11	0.127
Stride time, s	1.03 ± 0.27	0.99 ± 0.16	0.223
Step length, m	0.39 ± 0.11	0.45 ± 0.09	<0.001 *
Stride length, m	0.79 ± 0.22	0.88 ± 0.22	0.005 *
Walking speed, m/s	0.8 ± 0.25	0.89 ± 0.23	0.008 *

Values are given as mean ± standard deviation. *p < 0.05.

**Table 4:** Comparisons of kinematic parameters (degrees) between the groups.

	Group I	Group II	*p
Average anterior pelvic tilt	19.20 ± 6.04	15.76 ± 5.67	<0.001 *
Average pelvic rotation	−13.17 ± 5.75	−0.81 ± 3.77	<0.001 *
Pelvic rotation at initial contact	−8.45 ± 7.29	1.84 ± 5.47	<0.001 *
Average pelvic obliquity	−1.97 ± 4.5	−0.51 ± 2.68	0.004 *
Maximum hip extension	6.70 ± 11.32	−0.59 ± 7.98	<0.001 *
Average hip rotation	1.37 ± 9.86	−1.57 ± 8.04	0.017 *
Maximum knee flexion	41.55 ± 10.00	45.78 ± 9.20	0.002 *
Maximum ankle dorsiflexion	3.39 ± 13.48	10.46 ± 7.64	<0.001 *
Ankle dorsiflexion at initial contact	−11.16 ± 10.07	−6.90 ± 7.44	<0.001 *
Average ankle rotation	5.95 ± 14.10	−0.63 ± 10.43	<0.001 *

Values are given as mean ± standard deviation. *p < 0.05.

**Table 5:** Gait factors classified by clustering technique.

	Factor1	Factor2	Factor3	Factor4	Factor5	Factor6	Factor7	Factor8
Age	0.058	0.573	0.345	0.007	0.260	0.012	0.074	−0.052
Cadence	−0.055	−0.083	−0.962 *	0.005	0.041	−0.039	0.009	0.002
Stride time	−0.001	−0.004	0.993 *	0.019	−0.021	0.011	−0.028	−0.004
Step time	0.013	−0.008	0.962 *	0.030	−0.043	0.003	−0.014	0.025
Stride length	−0.017	0.922 *	0.164	−0.016	−0.052	0.045	−0.041	−0.001
Step length	−0.078	0.924 *	0.044	0.003	−0.042	−0.011	−0.062	−0.021
Walking speed	0.006	0.858 *	−0.323	−0.019	−0.066	0.045	0.001	0.017
ROM of pelvic sagittal motion	−0.261	−0.076	0.144	0.061	−0.181	0.013	0.372	0.168
Avg. pelvic anterior tilt	0.032	−0.074	−0.093	0.002	0.017	0.011	0.902 *	−0.032
ROM of pelvic coronal motion	0.003	0.232	−0.034	−0.043	−0.361	0.166	0.516	0.068
Avg. pelvic obliquity	−0.013	−0.130	0.039	0.182	0.205	0.736 *	−0.350	−0.197
Min. hip sagittal angle	−0.048	−0.104	0.136	−0.019	0.464	−0.038	0.641	−0.039
Avg. hip sagittal angle	0.012	0.027	−0.015	0.052	0.538	−0.034	0.678	−0.082
Max. hip coronal angle	−0.031	0.099	0.034	−0.035	−0.091	0.879 *	0.174	0.068
Avg. hip coronal angle	0.021	0.034	0.012	−0.056	0.056	0.944 *	0.089	0.018
Max. hip transverse angle	0.034	−0.001	−0.068	0.069	0.032	−0.057	0.005	0.940 *
Avg. hip transverse angle	0.014	−0.072	0.084	0.054	0.091	0.032	−0.009	0.929 *
Min. knee sagittal angle	−0.028	0.185	−0.082	−0.077	0.954 *	0.041	−0.119	0.046
ROM of knee sagittal motion	0.148	0.178	−0.129	0.175	−0.673	−0.007	0.039	−0.173
Knee sagittal angle at IC	0.137	−0.069	−0.203	0.287	0.525	0.100	0.171	−0.037
Avg. knee transverse angle	0.034	0.528	0.067	0.139	0.130	−0.418	0.046	−0.109
Max. ankle sagittal angle	0.935 *	−0.002	0.016	−0.016	−0.029	−0.003	−0.049	−0.013
Min. ankle sagittal angle	0.898 *	−0.059	0.126	−0.069	−0.067	−0.047	0.033	0.010
Avg. ankle sagittal angle	0.962 *	0.001	0.031	−0.038	−0.029	−0.008	0.009	0.008
Ankle sagittal angle at IC	0.949 *	−0.012	−0.067	0.046	0.016	0.038	−0.017	0.054
Avg. ankle transverse angle	−0.095	−0.156	0.089	0.846 *	−0.233	−0.018	0.079	−0.217
Max. foot progression angle	−0.047	0.089	−0.008	0.921 *	0.052	−0.006	−0.026	0.173
Avg. foot progression angle	0.024	0.074	−0.004	0.947 *	0.061	0.011	−0.043	0.162

* Factor > 0.7000. Avg.: average; Max.: maximum; Min.: minimum; ROM: range of motion; IC: initial contact.

**Table 6:** Multivariate logistic regression analysis with gait and clinical factors.

		Estimate	Standard Error	95% Wald		Pr > *k*^2^
				Confidence	Limits	
Factor 1 (Ankle dorsiflexion)		0.382	0.350	0.192	0.757	0.006 *
Factor 2 (Walking speed, stride & step length)		0.689	0.236	0.434	1.094	0.114
Factor 3 (Cadence, stride & step time)		1.013	0.219	0.660	1.555	0.953
Factor 4 (Internal rotation of foot and ankle)		0.935	0.236	0.589	1.484	0.776
Factor 5 (Knee extension)		1.411	0.332	0.736	2.706	0.299
Factor 6 (Pelvic obliquity and hip abduction)		1.037	0.257	0.627	1.715	0.889
Factor 7 (Anterior pelvic tilt)		2.305	0.263	1.376	3.861	0.002 *
Factor 8 (Hip internal rotation)		1.540	0.217	1.006	2.358	0.047 *
Modified Winters classification	5 vs 1	0.015	1.590	<0.001	0.841	0.021 *
	4 vs 1	0.844	0.584	0.190	3.754	0.548
	3 vs 1	1.540	0.552	0.452	5.253	0.084
	2 vs 1	3.785	0.658	1.202	11.921	0.005 *
Gastrocnemius tightness		1.001	0.034	0.936	1.070	0.986
Achilles tendon tightness		0.998	0.033	0.935	1.065	0.947
Pes equinovarus		2.381	0.266	0.838	6.763	0.103
Asymmetrical posturing of the upper extremity		3.440	0.295	1.081	10.941	0.036 *

* *k*^2^ < 0.05.
